# Editorial: Cooperative Adaptation and Evolution in Plant-Microbe Systems

**DOI:** 10.3389/fpls.2018.01090

**Published:** 2018-08-14

**Authors:** Tatiana Matveeva, Nikolai Provorov, Jari P. T. Valkonen

**Affiliations:** ^1^Department of Genetics and Biotechnology, St. Petersburg State University, St. Petersburg, Russia; ^2^All-Russia Research Institute for Agricultural Microbiology, St. Petersburg, Russia; ^3^Department of Agricultural Sciences, University of Helsinki, Helsinki, Finland

**Keywords:** plant-microbe interaction, coadaptation, coevolulion, horizontal gene transfer, ecological genetics, beneficial and antagonistic symbioses, signal-receptor interactions, symbiotic N_2_ fixation

Evolutionarily, plant-microbe interactions range from beneficial symbioses to the molecular arms race between pathogens and the immune systems of plants. Expanding our knowledge on ecological and evolutionary genetics of plant-microbe interactions is of high importance. Plants coevolve symbiotically with enormously diverse microbial communities, which has been pivotal since colonization of land by plants. The fungal and bacterial associates provide plants with important nutritional, protective and growth regulatory functions.

The well-studied mutualists (legume N_2_-fixing nodules, arbuscular mycorrhizae) and antagonists (biotrophic and necrotrophic) represent only a minority of symbioses between plants and associated microbial communities. The endophytic and epiphytic microbiomes exceed their hosts greatly in terms of genetic information potentially useful for extending the plant ecological amplitude and improving crop production.

A major breakthrough in conceptualizing the role of plant-microbe interactions in evolution has become possible largely owing to the new research methods. Next generation sequencing (NGS) opens up new prospects for studies in inter-species interactions. On the one hand, analysis of the accumulating data makes it possible to approach macroevolution from a new angle. The study presented by Wang et al. in this issue of Frontiers in Plant Science lays ground for discussions about evolution of stramenopiles and more complex scenarios for the evolution of oomycetes, including the supposed ancestral endosymbioses or independent horizontal gene transfer events involving red and green algae, oomycetes and other stramenopiles.

NGS provides opportunities for deeper study of genomes and transcriptomes of species, new gene combinations and differentially expressed genes during the symbiotic interactions. For instance, *Phomopsis liquidambari* studied by Zhou et al. is established in endophytic and saprophytic systems with rice (*Oryza sativa* L.). Most genes for amino acids and carbohydrate metabolism, fatty acid biosynthesis, and secondary metabolism are up-regulated in endophytic fungi. Most pathways of xenobiotic biodegradation and metabolism are upregulated in saprophytic systems, demonstrating the genetic regulation of adaptation to various ecological niches.

Symbiotic relationships contribute not only to changes in the pattern of gene expression, but also to the exchange of genes between symbionts. NGS helps to find footprints of such exchanges. Five different types of T-DNA of *Rhizobium rhizogenes* (formerly *Agrobacterium rhizogenes*) were identifed in *Nicotiana* during the analysis of genome sequence data, supplementing former information about T-DNA in *Nicotiana* and *Linaria* species with new types of T-DNA. Homologues of T-DNAs of agrobacteria were fortuitously found in the genome of sweetpotato while assembling small interfering RNAs (siRNAs) for metagenomic analysis. All this suggests that horizontal gene transfer between bacteria and plants is more widespread in evolution than previously thought (Chen and Otten; Quispe-Huamanquispe et al.). However, to understand the function of the transferred genes, additional studies are required.

Another interesting and actively studied aspect of the interaction of plants and rhizobia is nitrogen-fixing symbiosis. Evolution of N_2_-fixing symbioses may be addressed as a conduit of constructing N_2_-fixing plants. Construction of “ammonioplasts” by N_2_-fixing intracellular symbionts was reviewed by de la Peña et al. who outlined that symbiosomes of galegoid legumes represent functional and structural analogs of regular cellular organelles. Moreover, in some legumes, the symbiosome formation is compatible with the host cell divisions suggesting a possibility for regeneration of plants stably maintaining the ammonioplasts.

N_2_-fixing organelles can be constructed by introducing *nif* genes into regular organelles since their free-living ancestors probably possessed those genes. Burén et al. have demonstrated that the functional *nifB* may be expressed in mitochondria of yeast and tobacco, whereas Arragain et al. found broad phylogenetic diversity of *nifB* in thermo- and mesophylic microbes. Hence, it seems possible to select optimal *nifB* alleles for expression in eukaryotic cells.

For developing the novel N_2_-fixing systems, it is important to dissect the evolutionary mechanisms operating in the extant symbioses, wherein the evolution of N_2_-fixing bacteria is directed by plant hosts. An important approach is to analyze the evolution of plant receptor genes and signatures of different types of natural selection, characterized by nonsynonymous/synonymous nucleotide substitutions. Their functionality was demonstrated for the family of LysM-RLK receptors in *Pisum sativum* recognizing the *Rhizobium leguminosarum* Nod factors, since different selection types (purifying, positive, balancing) are attributed to the functionally diverse plant gene domains (Sulima et al.). Evolution of symbiotically specialized plant genes may be sufficiently intensified due to their cytokinin-dependent local and systemic regulation which was for the first time demonstrated in *Medicago truncatula* (Azarakhsh et al.).

An alternative, antagonistic strategy of plant-microbe interactions may be represented by defense against pathogens. Co-evolution of plants and pathogens has affected the various mechanisms of their interaction. Defense against viruses in plants is largely based on recognition and degradation of double-stranded viral RNA by a mechanism called RNA interference (RNAi). New plant genes that participate in the RNAi pathway and enhance its function are reported by Zhu et al. Plants undergo molecular arms race also with pathogens that interfere with other immune systems, e.g., by inhibiting signaling molecules such as reactive oxygen species (ROS) triggering defense responses upon infection. Jwa and Hwang provide updates of the mechanisms by which pathogen effectors interfere with ROS signaling in plants. Plants can also warn neighboring plants by signaling via volatile organic compounds (VOCs). The plants wounded by insects or pathogens produce VOCs triggering defense mechanisms in undamaged plants, induce root growth and enhance beneficial root microbes, and hence improve overall resistance of the plant to pathogens and pests (Sweeney et al.).

Besides the biotic stresses caused by pathogens and pests, plants face abiotic stresses such as drought and high or low temperatures that may be most limiting for growth. Non-pathogenic microbes may alleviate such stresses. Tiwari et al. report that a strain of *Bacillus amyloliquefaciens* living in the plant root system is able to reduce abiotic stresses in rice via cross-talk with pathways regulating stresses and phytohormones. Similarly, studies of Bilal et al. show that soil microbes such as *Paecilomyces formosus* can help alleviating the stress of plants caused by heavy metals such as nickel in plants.

The studies published in this thematic issue contribute to a broad range of fundamental and applied themes and integrate top level research on genetic interactions in plant-microbe associations. The novel information reported is required to develop the prospective evolutionary approaches for the study of molecular and ecological genetics of symbioses.

## Author contributions

TM, NP, and JV edited the topic and wrote an editorial.

### Conflict of interest statement

The authors declare that the research was conducted in the absence of any commercial or financial relationships that could be construed as a potential conflict of interest.

